# *Echinococcus canadensis* G8 Tapeworm Infection in a Sheep, China, 2018

**DOI:** 10.3201/eid2507.181585

**Published:** 2019-07

**Authors:** Ruiqi Hua, Yue Xie, Hongyu Song, Yuan Shi, Jiafei Zhan, Maodi Wu, Xiaobin Gu, Xuerong Peng, Guangyou Yang

**Affiliations:** Sichuan Agricultural University, Chengdu, China

**Keywords:** cystic echinococcosis, *Echinococcus canadensis*, parasites, G8 tapeworm, tapeworm, flatworm, *nad1*, *cox1*, host range, geographic distribution, China, *Ovis aries*, sheep, intermediate host, zoonoses

## Abstract

We report a sheep infected with *Echinococcus canadensis* G8 tapeworm in China in 2018. This pathogen was previously detected in moose, elk, muskox, and mule deer in Europe and North America; our findings suggest a wider host range and geographic distribution. Surveillance for the G8 tapeworm should be conducted in China.

Cystic echinococcosis (CE) is a zoonotic disease of worldwide distribution that causes disease, death, and economic loss in many domestic and wildlife ungulates and carnivore species, as well as in humans. Animals and humans can become infected through the accidental ingestion of *Echinococcus* tapeworm eggs (*1*,*2*). *Echinococcus granulosus* sensu stricto (G1, G3) tapeworms are considered the major cause of CE globally; however, cases attributable to *E*. *canadensis* genotypes within the *E*. *granulosus* tapeworm complex are increasingly being recognized (*3*). Overall, *E*. *canadensis* tapeworms comprise 4 genotypes (G6, G7, G8, G10), although the taxonomy is still being debated (*4*). *E*. *canadensis* G8 tapeworms were initially identified in 1994 in a moose (*Alces alces*) in Minnesota, USA (Appendix Table). Then, in 2002, two infections were reported in humans in Alaska. G8 tapeworms have also been found in elk (*Cervus canadensis*, 2006) and muskox (*Ovibos moschatus*, 2013) in Canada. Updated epidemiologic data show infections have also occurred in Estonia moose (2008), Russia moose (2013), and a US mule deer (*Odocoileus hemionus*, 2018). As of April 2019, at least 4 species (moose, elk, muskox, and mule deer) have been proven to serve as intermediate hosts of G8 tapeworms in Europe and North America. We report a potential new public health threat regarding sheep (*Ovis aries*) infected with *E*. *canadensis* G8 tapeworms in China and highlight the potential wider host range and geographic distribution of this species.

During 2017, we conducted a molecular epidemiologic survey of CE in northwestern China and collected 277 hydatid cysts from sheep (78 from Qinghai-Tibet Plateau, 60 from Xinjiang Autonomous Region) and yaks (*Bos mutus*; 139 from Qinghai-Tibet Plateau) at local slaughterhouses. During sampling, we handled all animals in strict accordance with the animal welfare laws of China. We genotyped the hydatid cysts using the partial mitochondrial *cox1* gene sequence, as described previously (*5*), and found that most cyst specimens were represented by *E*. *granulosus* G1 and G3 tapeworms (data not shown), and 1 sheep cyst was diagnosed as an *E*. *canadensis* G8–like tapeworm infection (herein designated sheep-XN) (Appendix Figure 1, panel A). To further investigate the genotype of tapeworm sheep-XN, we amplified the full-length *cox1* gene (1,608 bp) and the mitochondrial *nad1* gene (894 bp), a method proven effective for *Echinococcus* tapeworm genotyping (*4*). This analysis verified that sheep-XN clustered with *E*. *canadensis* G8 tapeworms (Appendix Figure 1, panel B). However, given that partial mitochondrial DNA (mtDNA) sequences are insufficient to identify genotype (because of limited loci information) (*6*), we amplified the complete mtDNA of sheep-XN and compared it with *Echinococcus* mtDNA sequences from GenBank. The resulting phylogenetic tree showed the same topologic structure as that acquired when using the *cox1* and *nad1* genes, suggesting that sheep-XN was an *E*. *canadensis* G8 tapeworm (Figure).

**Figure:**
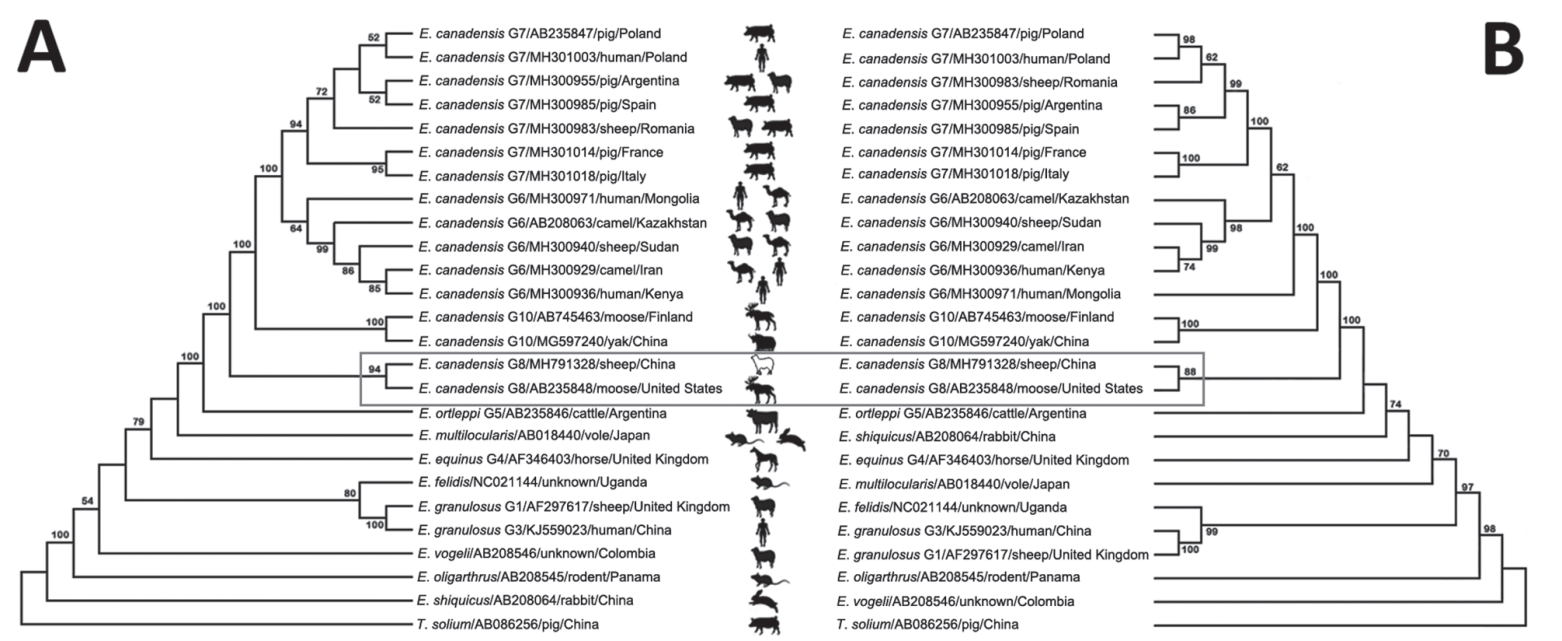
Phylogenetic analysis of *Echinococcus* species of different genotypes, strains, and host origins, including the *E*. *canadensis* G8 tapeworm identified in a sheep in China, 2018. Phylogenetic trees were inferred by maximum-likelihood analysis on the basis of concatenated amino acid data of 12 protein-coding genes by using the Jones-Taylor-Thornton model (A) and concatenated nucleotide data of 12 protein-coding genes by using the Tamura-Nei model (B) in MEGA7.0 (https://www.megasoftware.net). The reference species *Taenia solium* was used as the outgroup. We performed bootstrapping with 1,000 replicates to calculate the percentage reliability for each node in both data sets; only values of >50% are shown. Tree branch lengths are proportional to the evolutionary distance. The box contains the *E*. *canadensis* G8 tapeworm identified in this study (GenBank accession no. MH791328) and its closest relative from a moose in the United States (GenBank accession no. AB235848). Sheep shown in white represents a potential new intermediate host of *E*. *canadensis* G8.

We confirmed that the sheep-origin hydatid cyst was *E*. *canadensis* G8 tapeworm (Appendix Figure 1, panel C) and suggest that this pathogen potentially poses a new public health threat on the Qinghai-Tibet Plateau of China, where human echinococcosis is prevalent. Previous research has shown that sterile cysts usually result when *Echinococcus* spp. infect species not commonly infected (*7*). However, for the sheep-origin cyst, we found numerous protoscoleces in the hydatid fluid, indicating the cyst was fertile. Thus, sheep might serve as another intermediate host of the *E*. *canadensis* G8 tapeworm in nature and spread protoscoleces to definitive hosts, posing a threat to local herdsmen and livestock.

G6 and G7 tapeworms can circulate through the domestic cycle (in animals such as camels, pigs, and dogs) or the sylvatic cycle (in animals such as reindeer and wolves), and G8 and G10 tapeworms are generally believed to be restricted to the sylvatic cycle in circumpolar regions (Appendix Table) (*2*,*8*). Our finding of an *E*. *canadensis* G8 tapeworm in a sheep in China should not only alert the local population to be aware of this pathogen but also contributes to the discussion concerning *E*. *canadensis* tapeworm taxonomy. Further research is required to determine the transmission dynamics of this pathogen and determine whether the domestic life cycle of *E*. *canadensis* G8 tapeworm (circulation through sheep and dogs) has been or is present.

Since 2017, a mandatory vaccination campaign of sheep and goats with the CE vaccine EG95 has been sponsored in high-prevalence areas of China because of China’s policy, the National Medium- and Long-Term Plan for Animal Disease Control (2012–2020) (*9*). However, EG95 was developed against the *E*. *granulosus* G1 tapeworm (*10*) and might not provide effective protection against the *E*. *canadensis* G8 tapeworm. Our findings indicate the G8 tapeworm might be prevalent in sheep in China, suggesting a wider host range and geographic distribution (Appendix Table, Figure 2). Thus, we propose the need for increased surveillance of the *E*. *canadensis* G8 tapeworm in China and that integration of this pathogen into ongoing echinococcosis programs is essential for tapeworm prevention and control.

Appendix Additional information on *Echinococcus canadensis* G8 tapeworm infection in a sheep, China, 2018.
