# Supplementary material for: Echinococcus canadensis G8 Tapeworm Infection in a Sheep, China, 2018
Source: Emerg Infect Dis. 2019 Jul;25(7):1420–2. doi: 10.3201/eid2507.181585 (PMC6590743; doi:10.3201/eid2507.181585)
Supplement: Appendix — Additional information on Echinococcus canadensis G8 tapeworm infection in a sheep, China, 2018. [file 18-1585-Techapp-s1.pdf]

Article DOI: https://doi.org/10.3201/eid2507.181585

# *Echinococcus canadensis* G8 Tapeworm Infection in a Sheep, China, 2018

## Appendix

**Appendix Table.** The host range and geographic distribution of *Echinococcus canadensis* tapeworm, 1992–2018

| Genotype | Definitive hosts | Intermediate hosts | Geographic distribution | References |
|---|---|---|---|---|
| *E. canadensis* G6/7 | Dog, wolf | Camel, pig, cattle, goat, sheep, reindeer | Mexico, Peru, Brazil, Chile, Argentina, Tunisia, Algeria, Libya, Namibia, Mauritania, Ghana, Egypt, Sudan, Ethiopia, Somalia, Kenya, South Africa, Spain, Portugal, Poland, Ukraine, Czechia, Austria, Hungary, Romania, Serbia, Russia, Vatican City State, Bosnia and Herzegovina, Slovakia, France, Lithuania, Italy, Turkey, Iran, Afghanistan, India, Nepal, Kazakhstan, Kyrgyzstan, China, Mongolia | (*1*–*15*) |
| *E. canadensis* G8 | Wolf | Moose, elk, muskox, mule deer, sheep | America, Canada, Estonia, Latvia, Russia, China | |
| *E. canadensis* G10 | Dog, wolf | Moose, elk, reindeer, mule deer, yak | Finland, Mongolia, America, Canada, Estonia, Latvia, Sweden, Russia, China | |

### References


1. Moks E, Jõgisalu I, Valdmann H, Saarma U. First report of *Echinococcus granulosus* G8 in Eurasia and a reappraisal of the phylogenetic relationships of 'genotypes' G5-G10. Parasitology. 2008;135:647–54. PubMed http://dx.doi.org/10.1017/S0031182008004198

2. Nakao M, Lavikainen A, Yanagida T, Ito A. Phylogenetic systematics of the genus *Echinococcus* (Cestoda: Taeniidae). Int J Parasitol. 2013;43:1017–29. PubMed http://dx.doi.org/10.1016/j.ijpara.2013.06.002

3. Thompson RCA. Biology and systematics of *Echinococcus*. In: Thompson RCA, Deplazes P, Lymbery AJ, editors. Advanced parasitology. Vol. 95. San Diego: Elsevier Academic Press Inc.; 2017. p. 65–110.

4. Ito A, Nakao M, Lavikainen A, Hoberg E. Cystic echinococcosis: Future perspectives of molecular epidemiology. Acta Trop. 2017;165:3–9. PubMed http://dx.doi.org/10.1016/j.actatropica.2016.05.013

5. Addy F, Wassermann M, Kagendo D, Ebi D, Zeyhle E, Elmahdi IE, et al. Genetic differentiation of the G6/7 cluster of *Echinococcus canadensis* based on mitochondrial marker genes. Int J Parasitol. 2017;47:923–931. PubMed http://dx.doi.org/10.1016/j.ijpara.2017.06.003

6. Yanagida T, Lavikainen A, Hoberg EP, Konyaev S, Ito A, Sato MO, et al. Specific status of *Echinococcus canadensis* (Cestoda: Taeniidae) inferred from nuclear and mitochondrial gene sequences. Int J Parasitol. 2017;47:971–979. PubMed http://dx.doi.org/10.1016/j.ijpara.2017.07.001

7. Wu Y, Li L, Zhu G, Li W, Zhang N, Li S, et al. Mitochondrial genome data confirm that yaks can serve as the intermediate host of *Echinococcus canadensis* (G10) on the Tibetan Plateau. Parasit Vectors. 2018;11:166. PubMed http://dx.doi.org/10.1186/s13071-018-2684-0

8. Laurimäe T, Kinkar L, Romig T, Omer RA, Casulli A, Umhang G, et al. The benefits of analysing complete mitochondrial genomes: Deep insights into the phylogeny and population structure of *Echinococcus granulosus* sensu lato genotypes G6 and G7. Infect Genet Evol. 2018;64:85–94. PubMed http://dx.doi.org/10.1016/j.meegid.2018.06.016

9. Cerda JR, Ballweber LR. Confirmation of *Echinococcus canadensis* G8 and G10 in Idaho Gray Wolves (*Canis lupus*) and Cervids. J Wildl Dis. 2018;54:403–405. PubMed http://dx.doi.org/10.7589/2017-05-119

10. Bowles J, Blair D, McManus DP. Molecular genetic characterization of the cervid strain (‘northern form’) of *Echinococcus granulosus*. Parasitology. 1994;109:215–21. PubMed http://dx.doi.org/10.1017/S0031182000076332

11. Schurer J, Shury T, Leighton F, Jenkins E. Surveillance for *Echinococcus canadensis* genotypes in Canadian ungulates. Int J Parasitol Parasites Wildl. 2013;2:97–101. PubMed http://dx.doi.org/10.1016/j.ijppaw.2013.02.004

12. Nakao M, Yanagida T, Konyaev S, Lavikainen A, Odnokurtsev VA, Zaikov VA, et al. Mitochondrial phylogeny of the genus *Echinococcus* (Cestoda: Taeniidae) with emphasis on relationships among *Echinococcus canadensis* genotypes. Parasitology. 2013;140:1625–36. PubMed http://dx.doi.org/10.1017/S0031182013000565

13. Konyaev SV, Yanagida T, Nakao M, Ingovatova GM, Shoykhet YN, Bondarev AY, et al. Genetic diversity of *Echinococcus* spp. in Russia. Parasitology. 2013;140:1637–47. PubMed http://dx.doi.org/10.1017/S0031182013001340

14. McManus DP, Zhang L, Castrodale LJ, Le TH, Pearson M, Blair D. Short report: molecular genetic characterization of an unusually severe case of hydatid disease in Alaska caused by the cervid strain of *Echinococcus granulosus.* Am J Trop Med Hyg. 2002;67:296–8. PubMed http://dx.doi.org/10.4269/ajtmh.2002.67.296

15. Thompson RCA, Boxell AC, Ralston BJ, Constantine CC, Hobbs RP, Shury T, et al. Molecular and morphological characterization of *Echinococcus* in cervids from North America. Parasitology. 2006;132:439–47. PubMed http://dx.doi.org/10.1017/S0031182005009170

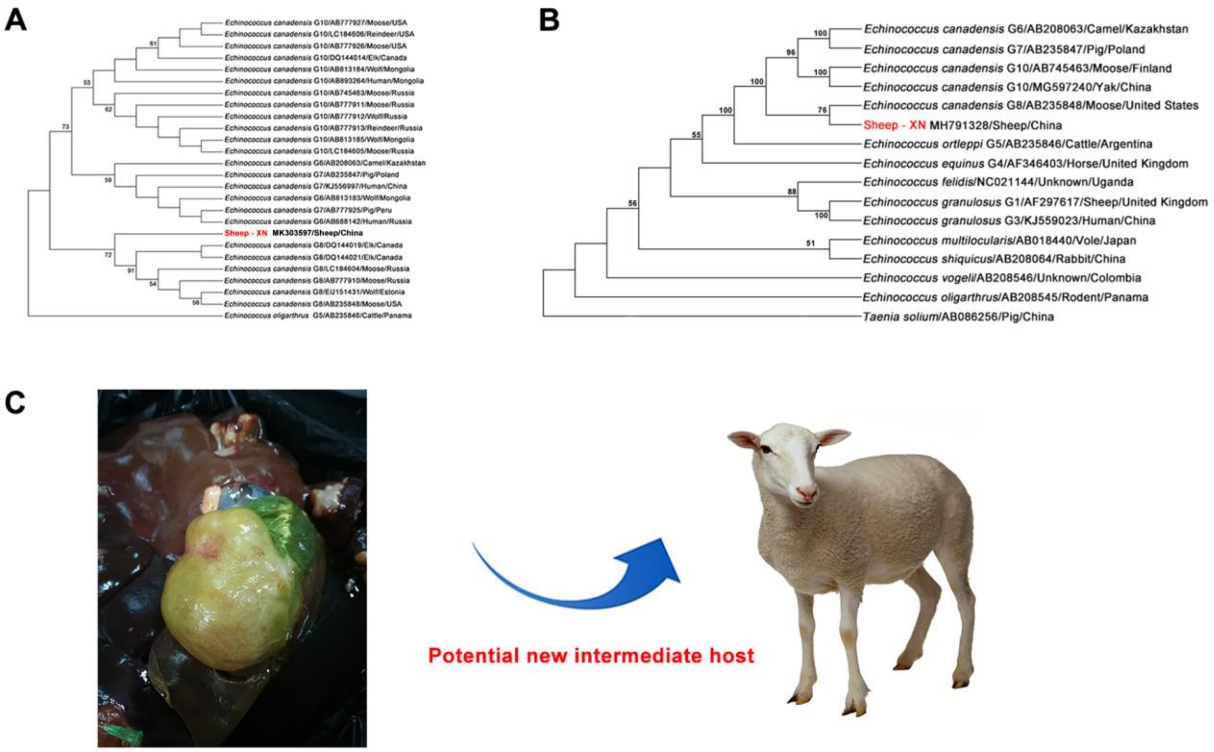


**Appendix Figure 1.** Molecular identification of *Echinococcus canadensis* G8 tapeworm from sheep in China, 2018. A) The phylogenetic tree was constructed by using the Kimura 2-parameter model based on maximum-likelihood analysis by using the partial sequences of mitochondrial *cox1* gene inferred from isolates of *Echinococcus* spp. *Echinococcus oligarthrus* was used as an outgroup. The maximum-likelihood tree was constructed by using MEGA7.0 (https://www.megasoftware.net). The bootstrap values >50% are shown for the nodes that appeared along the branches with 1,000 replicates. Most *Echinococcus* isolates used in this study represent the *Echinococcus granulosus* G1 or G3 clusters (data not shown), whereas 1 isolate (XN1, MK303597) from a sheep was identified as *Echinococcus canadensis* G8-like tapeworm and named after its host, Sheep-XN (shown in red). B) To further identify the genotype of Sheep-XN, the full-length *cox1* gene (1,608 bp) together with the complete mitochondrial *nad1* gene (894 bp) were amplified and concatenated to produce a robust maximum-likelihood tree by using the Kimura 2-parameter method. The bootstrap values >50% are shown for the nodes that appeared along the branches with 1,000 replicates. *Taenia solium* was used as the outgroup. These

results consistently showed that the genotype of Sheep-XN isolate is *E*. *canadensis* G8 tapeworm. C) Hydatid cyst on the sheep infected with Sheep-XN. XN, Xining.

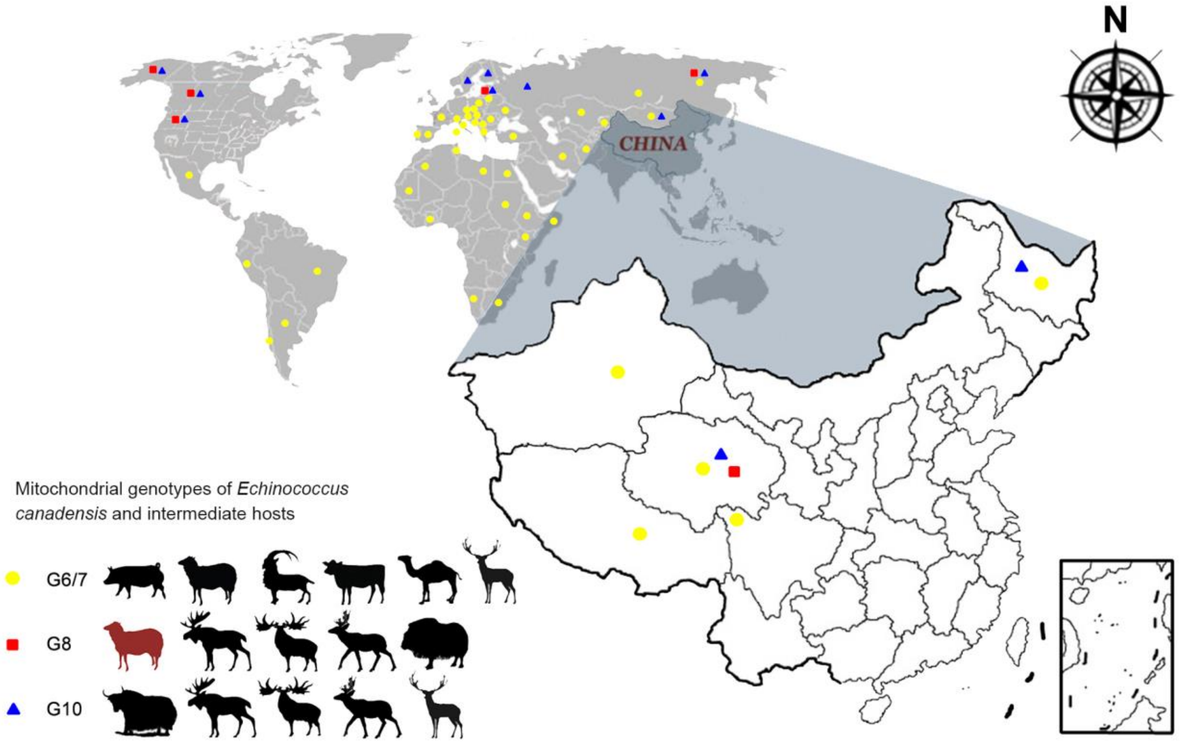


**Appendix Figure 2.** Global distribution of *Echinococcus canadensis* tapeworms based on mitochondrial data, with indications of intermediate host affiliation and emphasis on *Echinococcus canadensis* G8 tapeworm from sheep in China, 2018. The size of China is enlarged for easier visualization. The presence of *Echinococcus canadensis* G8 tapeworm in sheep from Xining (shown in red), located on the Qinghai-Tibet Plateau of China, suggests a wider intermediate host range and geographic distribution than previously acknowledged.
